# The perceptions and attitudes of medical students towards trauma and orthopaedic teaching: a cross-sectional study

**DOI:** 10.1051/sicotj/2016045

**Published:** 2017-02-10

**Authors:** Tarek Boutefnouchet, Basil Budair

**Affiliations:** 1 University Hospital Birmingham, Queen Elizabeth Hospital, Edgbaston Mindelsohn Way, Birmingham B15 2WB UK; 2 School of Medicine and Dentistry, The University of Birmingham, Edgbaston Birmingham B15 2TT UK

**Keywords:** Medical education, Undergraduate, Trauma, Orthopaedic, Survey

## Abstract

*Objectives*: This study aimed to identify how undergraduate students perceive learning opportunities available to them and to determine whether students with an interest in trauma and orthopaedic (T&O) surgery have different perceptions and attitudes towards learning.

*Methods*: All fourth year medical students from the University of Birmingham Medical School (UK) were surveyed regarding their career intentions and their attitudes towards the teaching received in trauma and orthopaedic surgery. The questionnaire was designed to capture student perception of learning environments, core knowledge and career motivations.

*Results*: Of the 157 respondents, 35 (22.3%) expressed an interest in a career in trauma and orthopaedic surgery. Medical students who reported educational value for trauma and orthopaedic surgery revealed that bedside teaching with a consultant was perceived extremely useful by 57.8% (*n* = 89). A similar ranking was awarded to small group teaching seminars and bedside teaching with a junior doctor or trainee by 54.5% (*n* = 85) and 51.6% (*n* = 79) of students, respectively. In contrast, trauma meetings and operating theatre learning environments were perceived to be of low educational value. Seeing patients within the clinical setting and the quality of teaching received were reported as the most motivating factors in career interest towards trauma and orthopaedic surgery, rated 43.9% (*n* = 69) and 35% (*n* = 55), respectively.

*Conclusions*: Perceptions of educational benefit derived from each learning environment vary among undergraduate medical students. Overall the most valuable learning environment perceived by the students is formal patient-based teaching. Despite diverging speciality choices students demonstrate similar learning needs.

## Introduction

Over one hundred years have elapsed since the milestone Flexner report and the establishment of a modern medical education curriculum [1]. Musculoskeletal teaching has been adversely affected by the discrepancy between the variety of clinical problems encountered in practice and medical schools curricula [2]. Bone and joint disorders remain a frequent health complaint, with back pain constituting a significant proportion of general practitioners’ workload. In the United Kingdom (UK), 10–25% of general practice consultations relate to musculoskeletal problems of which back pain constitutes up to a third [3]. In the United States (US), 10–18% of primary care consultations relate to musculoskeletal disorders with an annual estimated cost of $850 million (£530, €655) [4]. Undergraduate musculoskeletal education in the UK is often delivered in combination with other clinical placements and has to compete with other specialities in the curriculum [5]. The average undergraduate teaching time dedicated to trauma and orthopaedic (T&O) surgery is reported to be around two and a half weeks [6]. This is normally delivered within a five to eight week block combined with rheumatology, emergency medicine and other allied specialities [7]. This situation is echoed in the US where a 25-point objective examination introduced by Freedman and Bernstein was designed to assess undergraduate musculoskeletal knowledge [8]. A report from the Harvard Medical School demonstrated that only 26% of fourth year medical students passed this particular examination [9]. With the last decade being appointed the bone and joint decade [10], undergraduate musculoskeletal education, particularly T&O surgery, is in need of optimisation.

With the focus of medical education shifting towards continuous learning, students are encouraged to take more responsibility for their own training. Educational research has attempted to consider the question as to whether learning styles and strategies are adapted to conform to the chosen speciality. Recent evidence shows that career intent is present early on and is either nourished or ablated by external factors during early years of training [11]. Similarly, learning styles and personality types have a strong influence on career choices [12, 13]. It is therefore crucial to consider factors, which affect students when planning future educational developments. It is also undeniable that in order to improve curriculum design, students’ perception of learning opportunities and attitudes towards learning subjects must be considered.

The aim of this study was to investigate the perceived educational value of various learning exercises among fourth year medical students following their musculoskeletal clinical placement. This study evaluated the perceived usefulness of learning environments commonly encountered in the undergraduate trauma and orthopaedic curriculum. It also assessed the utility of these educational activities in relation to key topics in undergraduate musculoskeletal knowledge. Finally, the study hypothesised that medical students considering a career in trauma and orthopaedic surgery exhibit different perceived learning needs and attitudes towards the speciality.

## Methods

### Educational context

The undergraduate musculoskeletal curriculum at the University of Birmingham (UK) is delivered as one of the core clinical modules in the fourth year. Students undertake six core clinical modules throughout the academic year. These modules run in conjunction with the community medicine module, the teaching and learning module as well as student selected activities. Groups of students are allocated to a teaching hospital affiliated to the University medical school. The musculoskeletal component is spread over a block of five weeks with weekly scheduled lecture days delivered in a central university teaching hospital. During the remaining time the students are divided into subgroups spending a total of two weeks in trauma and orthopaedic surgery, two weeks in rheumatology and one week in emergency medicine. During those five weeks students are required to attend scheduled teaching sessions within their base hospital delivered predominantly by consultants, teaching fellows and other senior clinicians. Additional teaching is delivered in clinical areas including wards, outpatient clinics, daily trauma meetings and operating theatres. The topics covered during the placement are set out in the curriculum and learning objectives designed by the School of Medicine.

### Study design

In order to address the research questions a survey instrument was designed. A 5-point Likert scale was utilised in order to elicit students’ response to the perceived value of learning environments and attitudes towards key topics in musculoskeletal knowledge [14]. Content validity was assessed using the results of previous reports [15, 16]. Students were asked to rate their attitude towards learning environments encountered during their musculoskeletal module. The elements comprising the core knowledge of T&O surgery were based on the curriculum content. In addition, the core knowledge and key topics section of the questionnaire were selected due to their value as demonstrated in the literature [15–17]. The survey instrument comprised a question on whether students were interested in pursuing a career in T&O surgery. Factors, which motivated medical students towards this speciality as a career choice, were evaluated using the 5-point Likert scale.

### Study participants

Consecutive fourth year medical students from the University of Birmingham were asked to complete the survey. The inclusion period was July 2011 to June 2012 and the total cohort comprised 412 students. In terms of ethical considerations, the Medical School Education Unit provided institutional permission for the study. Student anonymity was maintained and questionnaires were not part of any institutional evaluation. Students were not offered incentives to participate and participation was not mandatory. We elected to carry out the survey at the end of the academic year in order to ensure capture of all students having completed their musculoskeletal module as well as avoiding interference with academic coursework. A website link was created for the survey questionnaire, and a link emailed to the students by the undergraduate school administrator. The survey was distributed over two rounds; the first returned 102 responses and the second 55 responses. A follow-up survey of non-responders was not performed because no identifying information was collected.

### Data collection and analysis

Results were initially collated online and tabulated using Excel Spread Sheet (Microsoft Excel Worksheet 2010^©^). Frequency distributions of the responses were tabulated and percentages were calculated based on the total number of respondents to each question. Data was then analysed using SPSS software, version 16.0 (SPSS Inc., Chicago, IL). Students’ interest in the speciality was measured as a binary variable. All analyses compared the difference between the two groups. The first set of variables related to the educational value given to each learning environment, and due to the ordinal nature of the scale, the Mann-Whitney test was used. Students’ perceptions of learning environments were measured on a categorical scale, with Fisher’s exact test used for the analyses. The final set of variables measured factors that influenced students’ interest; the Mann-Whitney test was used for the analysis. Significance level was set at 0.05.

## Results

One hundred and fifty-seven students completed the questionnaire with a total response rate of 40%. The perceived educational value towards learning in trauma and orthopaedic surgery revealed that bedside teaching with a consultant was considered extremely useful by 57.8% (*n* = 89). A similar ranking was awarded to small group teaching seminars and bedside teaching with a junior doctor or a trainee (54.5% (*n* = 85) and 51.6% (*n* = 79), respectively). Learning environments considered to be useful for this subject were scored as follows: formal lectures 51.6% (*n* = 81), seeing patients in outpatient clinics 48.4% (*n* = 76), seeing patients on the ward 47.4% (*n* = 73) and independent reading 45.9% (*n* = 72). The educational benefit of watching and assisting surgery revealed that 41.7% (*n* = 65) students did not find arthroscopic surgery useful compared to 14.1% (*n* = 22) of students who did find it useful. In addition, the educational value of the morning trauma meeting showed that only 16% (*n* = 25) found it useful compared to 31.4% (*n* = 49) who rated it as not useful. Table 1 provides a detailed outline of how students assessed the educational value for each learning environment. In addition, students found the greatest educational benefit for learning practical skills through seeing patients in the emergency department. Table 2 outlines how students rated the educational benefit of different learning environments in relation to musculoskeletal curriculum items.

**Table 1.** Trauma and orthopaedic surgery student assessed educational value for each learning environment.

Total *n* = 157	1	2	3	4	5	Median response	IQR	*P*-value Mann-Whitney
Interested in T&O career *n* = 35								
Not interested in T&O career *n* = 122								
Seeing patients in clinic	**1.3% (2)**	**5.7% (9)**	**15.9% (25)**	**48.4% (76)**	**28.7% (45)**	4	4–5	0.05
Interested in T&O career	0.0% (0)	0.0% (0)	11.4% (4)	51.4% (18)	37.1% (13)	4	4–5	
Not interested in T&O career	1.6% (2)	7.4% (9)	17.2% (21)	47.5% (58)	26.2% (32)	4	4–5	
Seeing patients on the ward	**3.2% (5)**	**11.7% (18)**	**26.6% (41)**	**47.4% (73)**	**11.0% (17)**	4	3–4	0.11
Interested in T&O career	0.0% (0)	8.6% (3)	20.0% (7)	60.0% (21)	11.4% (4)	4	3–4	
Not interested in T&O career	4.2% (5)	12.6% (15)	28.6% (34)	43.7% (52)	10.9% (13)	4	3–4	
Bedside teaching with SPR/SHO/FY*	**2.6% (4)**	**2.0% (3)**	**9.2% (14)**	**34.6% (53)**	**51.6% (79)**	5	4–5	0.33
Interested in T&O career	0.0% (0)	0.0% (0)	11.8% (4)	29.4% (10)	58.8% (20)	5	4–5	
Not interested in T&O career	3.4% (4)	2.5% (3)	8.4% (10)	36.1% (43)	49.6% (59)	5	4–5	
Bedside teaching with Consultant	**1.9% (3)**	**1.9% (3)**	**9.7% (15)**	**28.6% (44)**	**57.8% (89)**	5	4–5	0.11
Interested in T&O career	0.0% (0)	0.0% (0)	14.3% (5)	14.3% (5)	71.4% (25)	5	4–5	
Not interested in T&O career	2.5% (3)	2.5% (3)	8.4% (10)	32.8% (39)	53.8% (64)	5	4–5	
Watching/assisting open surgery	**11.5% (18)**	**21.7% (34)**	**36.3% (57)**	**21.0% (33)**	**9.6% (15)**	3	3–4	<0.001
Interested in T&O career	5.7% (2)	11.4% (4)	28.6% (10)	31.4% (11)	22.9% (8)	4	3–4	
Not interested in T&O career	13.1% (16)	24.6% (30)	38.5% (47)	18.0% (22)	5.7% (7)	3	3–4	
Watching/assisting arthroscopic surgery	**12.2% (19)**	**41.7% (65)**	**30.1% (47)**	**14.1% (22)**	**1.9% (3)**	2	2–3	0.40
Interested in T&O career	11.4% (4)	34.3% (12)	37.1% (13)	14.3% (5)	2.9% (1)	3	3–4	
Not interested in T&O career	12.4% (15)	43.8% (53)	28.1% (34)	14.0% (17)	1.7% (2)	2	2–3	
Morning trauma meeting	**17.3% (27)**	**31.4% (49)**	**28.8% (45)**	**16.0% (25)**	**6.4% (10)**	3	3–4	0.43
Interested in T&O career	11.4% (4)	34.3% (12)	28.6% (10)	17.1% (6)	8.6% (3)	3	3–4	
Not interested in T&O career	19.0% (23)	30.6% (37)	28.9% (35)	15.7% (19)	5.8% (7)	3	3–4	
Small group teaching seminar	**1.9% (3)**	**0.6% (1)**	**4.5% (7)**	**38.5% (60)**	**54.5% (85)**	5	4–5	0.64
Interested in T&O career	0.0% (0)	0.0% (0)	2.9% (1)	48.6% (17)	48.6% (17)	4	4–5	
Not interested in T&O career	2.5% (3)	0.8% (1)	5.0% (6)	35.5% (43)	56.2% (68)	5	4–5	
Formal lectures	**0.6% (1)**	**3.2% (5)**	**17.8% (28)**	**51.6% (81)**	**26.8% (42)**	4	4–5	0.52
Interested in T&O career	0.0% (0)	2.9% (1)	17.1% (6)	48.6% (17)	31.4% (11)	4	4–5	
Not interested in T&O career	0.8% (1)	3.3% (4)	18.0% (22)	52.5% (64)	25.4% (31)	4	4–5	
Independent reading	**1.3% (2)**	**7.0% (11)**	**21.7% (34)**	**45.9% (72)**	**24.2% (38)**	4	4–5	0.23
Interested in T&O career	2.9% (1)	11.4% (4)	22.9% (8)	42.9% (15)	20.0% (7)	4	4–5	
Not interested in T&O career	0.8% (1)	5.7% (7)	21.3% (26)	46.7% (57)	25.4% (31)	4	4–5	

* SPR: specialist registrar (equivalent senior resident). SHO: senior house officer (equivalent junior resident). FY: foundation year (equivalent internship). IQR: interquartile range. %: Percentage response to each question.

Question 1: How *useful* do you find each learning environment for trauma and orthopaedic surgery? (1 Not at all useful – 5 Extremely useful).

**Table 2.** Student rated educational benefit of learning environments in relation to key musculoskeletal curricular items.

Total *n* = 157	Seeing patients in ward/clinics	Attending operating lists	Formal teaching sessions	Morning trauma meetings	Seeing patients in A&E	*P*-value Fisher’s test
Interested in T&O career *n* = 35						
Not interested in T&O career *n* = 122						
Diagnosis and management of joint conditions e.g. OA, RA	**59.2% (93)**	**0.6% (1)**	**40.1% (63)**	**0.0% (0)**	**0.0% (0)**	0.25
Interested in T&O career	71.4% (25)	0.0% (0)	28.6% (10)	0.0% (0)	0.0% (0)	
Not interested in T&O career	55.7% (68)	0.8% (1)	43.4% (53)	0.0% (0)	0.0% (0)	
Diagnosis and management of Back pain	**44.6% (70)**	**0.0% (0)**	**50.3% (79)**	**0.6% (1)**	**4.5% (7)**	0.25
Interested in T&O career	37.1% (13)	0.0% (0)	54.3% (19)	2.9% (1)	5.7% (2)	
Not interested in T&O career	46.7% (57)	0.0% (0)	49.2% (60)	0.0% (0)	4.1% (5)	
Management of common fractures (Hip, ankle, wrist)	**21.7% (34)**	**11.5% (18)**	**32.5% (51)**	**7.6% (12)**	**26.8% (42)**	0.60
Interested in T&O career	22.9% (8)	8.6% (3)	25.7% (9)	5.7% (2)	37.1% (13)	
Not interested in T&O career	21.3% (26)	12.3% (15)	34.4% (42)	8.2% (10)	23.8% (29)	
Management of polytrauma e.g. open fractures	**6.4% (10)**	**10.2% (16)**	**51.0% (80)**	**9.6% (15)**	**22.9% (36)**	0.28
Interested in T&O career	8.6% (3)	17.1% (6)	37.1% (13)	11.4% (4)	25.7% (9)	
Not interested in T&O career	5.7% (7)	8.2% (10)	54.9% (67)	9.0% (11)	22.1% (27)	
Management of spinal and head Injuries	**4.5% (7)**	**1.3% (2)**	**68.2% (107)**	**3.2% (5)**	**22.9% (36)**	0.70
Interested in T&O career	8.6% (3)	0.0% (0)	65.7% (23)	2.9% (1)	22.9% (8)	
Not interested in T&O career	3.3% (4)	1.6% (2)	68.9% (84)	3.3% (4)	23.0% (28)	
Diagnosis and management of bone and soft tissue tumours	**17.2% (27)**	**3.2% (5)**	**74.5% (117)**	**1.3% (2)**	**3.8% (6)**	0.64
Interested in T&O career	20.0% (7)	0.0% (0)	74.3% (26)	2.9% (1)	2.9% (1)	
Not interested in T&O career	16.4% (20)	4.1% (5)	74.6% (91)	0.8% (1)	4.1% (5)	
Diagnosis and management of bone and joint infections	**12.7% (20)**	**2.5% (4)**	**79.6% (125)**	**1.9% (3)**	**3.2% (5)**	0.61
Interested in T&O career	14.3% (5)	2.9% (1)	74.3% (26)	2.9% (1)	5.7% (2)	
Not interested in T&O career	12.3% (15)	2.5% (3)	81.1% (99)	1.6% (2)	2.5% (3)	
X-ray interpretation	**24.2% (38)**	**5.1% (8)**	**35.0% (55)**	**26.1% (41)**	**9.6% (15)**	0.35
Interested in T&O career	34.3% (12)	2.9% (1)	31.4% (11)	28.6% (10)	2.9% (1)	
Not interested in T&O career	21.3% (26)	5.7% (7)	36.1% (44)	25.4% (31)	11.5% (14)	
Joints and spine examination	**51.0% (80)**	**0.6% (1)**	**42.7% (67)**	**0.6% (1)**	**5.1% (8)**	0.75
Interested in T&O career	45.7% (16)	0.0% (0)	51.4% (18)	0.0% (0)	2.9% (1)	
Not interested in T&O career	52.5% (64)	0.8% (1)	40.2% (49)	0.8% (1)	5.7% (7)	
Reducing dislocated joint/displaced fracture	**8.3% (13)**	**8.9% (14)**	**29.3% (46)**	**0.6% (1)**	**52.9% (83)**	0.30
Interested in T&O career	2.9% (1)	11.4% (4)	20.0% (7)	0.0% (0)	65.7% (23)	
Not interested in T&O career	9.8% (12)	8.2% (10)	32.0% (39)	0.8% (1)	49.2% (60)	
Applying a cast/plaster of Paris	**26.8% (42)**	**7.0% (11)**	**9.6% (15)**	**0.6% (1)**	**56.1% (88)**	0.23
Interested in T&O career	37.1% (13)	0.0% (0)	8.6% (3)	0.0% (0)	54.3% (19)	
Not interested in T&O career	23.8% (29)	9.0% (11)	9.8% (12)	0.8% (1)	56.6% (69)	
Joint injection/aspiration	**68.8% (108)**	**7.6% (12)**	**11.5% (18)**	**0.0% (0)**	**12.1% (19)**	0.40
Interested in T&O career	62.9% (22)	8.6% (3)	8.6% (3)	0.0% (0)	20.0% (7)	
Not interested in T&O career	70.5% (86)	7.4% (9)	12.3% (15)	0.0% (0)	9.8% (12)	

%: Percentage response to each question, OA: osteoarthritis, RA: rheumatoid arthritis, T&O: trauma and orthopaedic.

Question 2: In which learning environment did you *best* cover the following topics? (Please choose one BEST ANSWER for each topic).

Of the 157 respondents, 22.3% (*n* = 35) expressed their interest in trauma and orthopaedic surgery as a career choice. Seeing patients within the clinical setting 43.9% (*n* = 69) and the quality of teaching received 35% (*n* = 55) were reported as the most motivating factors in developing a specialist career interest. Seeing patients, quality of teaching received, assisting surgery and subject matter were ranked the most significant motivating factors. Table 3 outlines details of the results with scores in each motivating factor as ranked by the student responses. Our results demonstrate that watching or assisting in open surgery was significantly associated with the speciality interest. Over half (54%) of students who were interested in a T&O career gave a response of 4 or 5 to this category compared to only 24% who were not interested in a T&O career (Mann-Whitney test *p* < 0.001). There was also evidence that seeing patients in clinic was associated with being interested in a career in T&O surgery based on the ranking of motivating factors by students (Fisher’s exact test *p* < 0.001). In contrast, students’ responses indicated that the learning environment in relation to key curricular items was not significantly associated with being interested in the speciality. The most significant association was noted in direct patient contact, watching or assisting surgery and subject matter (Table 3).

**Table 3.** Motivating factors towards a career in trauma and orthopaedic surgery.

Total *n* = 157	1	2	3	4	5	Median response	IQR	*P*-value Mann-Whitney
Interested in T&O career *n* = 35								
Not interested in T&O career *n* = 122								
Seeing patients in clinic/ward/A&E	**6.4% (10)**	**9.6% (15)**	**15.9% (25)**	**43.9% (69)**	**24.2% (38)**	4	4–5	<0.001
Interested in T&O career	0.0% (0)	2.9% (1)	2.9% (1)	40.0% (14)	54.3% (19)	5	4–5	
Not interested in T&O career	8.2% (10)	11.5% (14)	19.7% (24)	45.1% (55)	15.6% (19)	4	3–4	
Quality of teaching received during placement	**8.3% (13)**	**11.5% (18)**	**28.0% (44)**	**35.0% (55)**	**17.2% (27)**	4	3–4	0.06
Interested in T&O career	2.9% (1)	5.7% (2)	25.7% (9)	45.7% (16)	20.0% (7)	4	3–4	
Not interested in T&O career	9.8% (12)	13.1% (16)	28.7% (35)	32.0% (39)	16.4% (20)	4	3–4	
Watching/assisting T&O surgery	**26.1% (41)**	**24.8% (39)**	**17.8% (28)**	**17.2% (27)**	**14.0% (22)**	2	2–3	<0.001
Interested in T&O career	5.7% (2)	11.4% (4)	11.4% (4)	25.7% (9)	45.7% (16)	4	4–5	
Not interested in T&O career	32.0% (39)	28.7% (35)	19.7% (24)	14.8% (18)	4.9% (6)	2	2–3	
Subject matter	**11.5% (18)**	**13.4% (21)**	**23.6% (37)**	**36.9% (58)**	**14.6% (23)**	4	3–4	<0.001
Interested in T&O career	0.0% (0)	2.9% (1)	8.6% (3)	45.7% (16)	42.9% (15)	4	4–5	
Not interested in T&O career	14.8% (18)	16.4% (20)	27.9% (34)	34.4% (42)	6.6% (8)	3	3–4	
T&O lifestyle	**38.9% (61)**	**25.5% (40)**	**22.9% (36)**	**11.5% (18)**	**1.3% (2)**	2	1–2	0.004
Interested in T&O career	20.0% (7)	28.6% (10)	28.6% (10)	20.0% (7)	2.9% (1)	3	3–4	
Not interested in T&O career	44.3% (54)	24.6% (30)	21.3% (26)	9.0% (11)	0.8% (1)	2	1–2	
T&O role models	**29.3% (46)**	**28.7% (45)**	**18.5% (29)**	**14.6% (23)**	**8.9% (14)**	2	2–3	0.002
Interested in T&O career	20.0% (7)	14.3% (5)	22.9% (8)	20.0% (7)	22.9% (8)	3	3–4	
Not interested in T&O career	32.0% (39)	32.8% (40)	17.2% (21)	13.1% (16)	4.9% (6)	2	2–3	

%: Percentage response to each question, T&O: trauma and orthopaedics, IQR: interquartile range.

Question 3: Which of the following makes you *interested* in trauma and orthopaedic surgery (T&O) as a possible career choice? (1 Not at all interested – 5 Extremely interested).

## Discussion

In order to maintain an optimal future output of competent orthopaedic surgeons, attention needs to be given to medical student education and curriculum design. The British Orthopaedic Association has issued a document in 2014 which outlines the expected level of knowledge and competencies newly qualified doctors should aim to achieve [18]. This highlights the importance of attention to be awarded to the field of musculoskeletal medicine within undergraduate curricula. Our study aimed to investigate the perceived educational value of various learning exercises among fourth year medical students following their musculoskeletal clinical placement. It looked at the efficacy of learning environments commonly encountered in the undergraduate trauma and orthopaedic curriculum. The present study demonstrated that consultant-led bedside teaching followed by small group teaching seminars were perceived to be the most beneficial, while the interaction with patients on wards and in clinics along with formal teaching sessions contributed the most to the acquisition of core orthopaedic knowledge. An interest in orthopaedic surgery as a future career was reported by a quarter of the respondents who perceived in-theatre experience and managing patients in clinic settings to be very valuable. The best-ranked learning environment among students interested in trauma and orthopaedics was bedside teaching with a consultant. Within this group core knowledge of musculoskeletal conditions and key clinical skills was best covered by seeing patients on the ward or emergency department and formal teaching sessions.

Multiple studies from across the world published in the last two decades show significant deficiencies in the undergraduate curriculums in covering essential musculoskeletal topics and call for improvements [8, 17, 19–26]. These shortcomings are reflected by the poor performance of fresh trauma and orthopaedic trainees, as demonstrated by a validated basic musculoskeletal competency examination with a failure rate of 82%, in the United States. Only those who had undertaken an additional undergraduate elective orthopaedic placement managed to score a pass mark in the examination [17]. To further emphasise their findings, the same authors reused the test after it was adjusted by a cohort of senior internal medicine physicians and it was equally associated with a high (78%) failure rate, thus indicating the inadequacy of undergraduate musculoskeletal education [8]. Skelly et al. and Matzkin et al., both from the United States, Queally et al., from Ireland, and Menon and Patro, from India, assessed medical students, general physicians and orthopaedic residents, respectively, and reported similar results with those who had undertaken an orthopaedic postgraduate rotation scoring significantly better, thus suggesting the importance of such placements [15, 23–25].

The situation in the UK is no different. In 2015, Al-Nammari et al. used the Freedman and Bernstein musculoskeletal cognitive examination tool to assess 200 and 10 medical students, who had already passed their final examinations [27]. Despite all students undertaking the mandatory orthopaedic rotation, with a mean duration of 2.65 weeks, and an additional 2.5 weeks in rheumatology in 96% of students, only 21% achieved a pass mark. Students with career interests in musculoskeletal specialities scored significantly higher, and those with interests in a career in general practice scored significantly lower than the remainder groups. A previous study from the same group reported the assessment of 112 junior doctors after completion of two-year foundation training in the UK and only 8.9% passed the assessment. The scores followed a similar pattern based on the doctors’ career intentions [28].

There is a global consensus on the limited access to musculoskeletal clinical teaching, which has an equal impact on graduates regardless of whether or not they are pursuing a future orthopaedic career. In addition, medical students need to spend more time in trauma and orthopaedic learning environments. Ali and Bulstrode showed a substantial variation in opinions about the amount of time needed to obtain basic trauma and orthopaedic knowledge and skills with orthopaedic surgeons suggesting that as much as eight weeks of dedicated orthopaedic teaching should be allocated in the medical school curricula [29]. However, we believe that a holistic approach to maximise the use of all potential educational opportunities is key to improve the quality of time spent in orthopaedic rotations. As previously reported, increasing lecture time may not improve medical students’ musculoskeletal knowledge [30] while learning orthopaedic topics using Podcasts showed a significantly higher satisfaction and gain of knowledge compared to conventional methods [31].

Our study highlights areas of missed educational opportunities for undergraduate students such as morning trauma meetings and in the operating theatres. This is also a concern highlighted by Dash et al. who showed that only 31% of surveyed foundation trainees in the UK attributed their limited theatre exposure to the fact that they lacked interest in surgical specialities [32]. The findings in our study were obtained from a cross-sectional survey of students in the same year and the same medical school. This carries some restrictions and may not be representative of students at different levels of training or those studying in other institutions, which might be implementing different curricula; but it ultimately echoes the findings published by many other studies from countries around the world. Our study stands out by showing that students with an interest in a career in trauma and orthopaedics share the same attitudes to knowledge acquisition and perception of educational value as those with no interest in an orthopaedic career. They do, however, have different motivational factors to pursuing a career in this speciality.

In this study, a number of potential limitations are present. Students may have a recollection bias when asked to recall the depth, usefulness and relevance of past teaching. Despite the two rounds, response rates from medical students were limited. This may be due to “questionnaire overload’’ which may have reduced response rates. The question on career choice was limited considering the vast array of career pathways a doctor can follow. The authors recognise that other speciality choices might have implications on students’ perceptions and attitudes towards learning musculoskeletal curriculum. However, some limitation on choices is needed for such a survey and crude estimates such as these can still help career planning. The causes of differences that exist between students’ desires and expectations on career progression were beyond the scope of this study. The results from this study should be generalised to other populations with caution. Our sample included only undergraduate students and as such may not represent the wider population. Although other educators may expect similar results from their students, some variability due to different teaching programmes and structures is to be expected. Finally, this study represents a descriptive report and not an interventional report. Controlled interventions with adequate endpoints in musculoskeletal teaching are lacking; this study however provides solid background information for future research.

## Conclusions

In an era of abundant service pressure, training in certain clinical areas, which were traditionally considered beneficial to undergraduate students, may need to be re-evaluated. Careful planning of targeted teaching in a suitable learning environment is still required. Core knowledge and key skills in trauma and orthopaedic surgery are learned through direct contact with patients. The discrepancy of what is perceived of educational value by students and educators needs to be elucidated through future research. The present study showed that regardless of whether or not a student is interested in pursuing a career in the speciality they have similar learning needs and similar perceptions of the most important learning activities. These results have implications in future curriculum design and delivery as well as selection of future orthopaedic surgeons.

## Conflict of interest

No competing interests declared, no external or commercial funds received by any of the authors.
